# Physical and Sociodemographic Features Associated With Quality of Life Among Transgender Women and Men Using Gender-Affirming Hormone Therapy

**DOI:** 10.3389/fpsyt.2021.621075

**Published:** 2021-07-26

**Authors:** Eliane D. Silva, Tayane M. Fighera, Roberta M. Allgayer, Maria Inês R. Lobato, Poli Mara Spritzer

**Affiliations:** ^1^Gynecological Endocrinology Unit, Division of Endocrinology, Hospital de Clínicas de Porto Alegre, Porto Alegre, Brazil; ^2^Gender Identity Program, Hospital de Clínicas de Porto Alegre, Porto Alegre, Brazil; ^3^Department of Physiology, Federal University of Rio Grande do Sul, Porto Alegre, Brazil

**Keywords:** transgender, cross-sex hormone therapy, quality of life, gender incongruence, gender dysphoria, gender-affirming hormone therapy

## Abstract

**Background:** Gender dysphoria is defined as a feeling of distress resulting from the incongruence between the sex assigned at birth and the gender identity, lasting longer than 6 months. In individuals with gender dysphoria, gender-affirming hormone therapy (GAHT) may improve quality of life (QoL).

**Objectives:** We aimed to assess perceived QoL, to compare QoL scores between trans women and men and to identify possible contributing factors related to GAHT in a sample of transgender women and transgender men.

**Methods:** In this cross-sectional study, transgender women and men were recruited by availability sampling from a national transgender health service. Individuals over 18 years old with a confirmed diagnosis of gender dysphoria receiving medically prescribed GAHT for at least 6 months were consecutively included. Also included were trans men who had undergone mastectomy and trans women who had received breast augmentation surgery. Individuals who had undergone gender affirmation surgery (specifically genital surgery) or with uncontrolled clinical/psychiatric conditions at the time of the initial assessment were excluded. Sociodemographic, physical, and hormone data were collected from all participants. The WHOQOL-BREF questionnaire was used to evaluate QoL. A total of 135 transgender individuals were invited. Seventeen individuals with previous genital surgery (12.6%) and five who refused to participate (3.7%) were excluded. Therefore, 113 patients were enrolled and completed the study (60 trans women and 53 trans men).

**Results:** QoL scores did not differ between trans women and trans men. In trans women, greater breast development and stable relationships, and higher body mass index were associated with higher QoL domain scores. In trans men, higher domain scores were found in individuals in a stable relationship, with increased body hair, engaging in physical activity, and being employed.

**Conclusion:** Data from this study suggest that GAHT-related physical characteristics, such as breast development in trans women and increased body hair in trans men, are similar between groups, are associated with higher QoL scores, and that sociodemographic parameters may impact these associations. Healthcare providers might consider these factors when planning interventions to improve QoL in transgender individuals.

## Introduction

Transgender is a term used to describe the incongruence between the gender identity and the sex assigned at birth (1). The wish to live and be accepted as a person of the opposite gender may be accompanied by a feeling of inadaptation and a desire to modify the body as much as possible into the gender identity (2) and is often associated with distress or dysphoria.

Although derived from limited data and possibly underestimated, the prevalence of gender incongruence has been reported as 4.6/100,000 individuals (6.8 for trans women and 2.6 for trans men) (3). More recent data from the United States show a prevalence of gender dysphoria of 390/100,000 individuals, or almost 1 million adults nationally (4).

Gender-affirming hormone therapy (GAHT) is often the first medical intervention used to relieve psychological suffering, minimize psychiatric comorbidities, and improve quality of life (QoL) in individuals with gender dysphoria (5). In GAHT, sexual hormones are used for development of secondary sex characteristics compatible with the gender identity and to reduce clinical characteristics of the birth gender (6). Trans women use oral or transdermal estrogen associated with antiandrogens, while trans men use injectable or transdermal testosterone (5, 6). GAHT initiation requires careful clinical and laboratory evaluation. Doses and administration routes vary according to the individual response and the clinical condition of each subject (6). The external physical changes induced by GAHT produce positive psychological effects, increase self-confidence, and facilitate conviviality and social interaction. In addition, GAHT-associated changes reinforce gender affirmation and social recognition (7–9).

According to the World Health Organization (WHO), QoL is a broad concept, encompassing the complex interplay between physical health, psychological status, level of independence, social relationships, personal beliefs, and the relationship with the environment (10–12). Individuals with gender dysphoria are more likely than cisgender individuals to experience discrimination, in both their personal and/or social life; this unequal treatment has the potential to affect all aspects of life, including the physical, psychological and well-being domains, as well as access to services and basic human rights (13). Previous studies report that factors such as gender affirmation surgery (GAS) (14), family support, working or studying (15) and body image can improve the QoL of transgender individuals (16).

The available data are controversial when comparing QoL between transgender individuals and the general population. While some studies have found no differences (17, 18), others have found that transgender individuals have lower physical and mental QoL scores compared with a control group (19, 20). Also, previous studies have shown that trans men have better QoL scores than trans women on the physical functioning subscale (20, 21). Changes induced by testosterone therapy, including body and facial hair growth, changes in body composition, and voice deepening, may contribute to the satisfaction of these individuals (6, 22, 23).

In addition, most QoL studies related to gender dysphoria to date have addressed the results of gender affirmation surgery (GAS), only a few have evaluated the impact of clinical changes resulting from GAHT on QoL parameters. Previous data suggest that GAHT has a positive effect on well-being (8) health in general, self-esteem (24), anxiety, depression (25), cognitive function (26), and QoL (15, 27). Recently, a systematic review including 7 observational studies assessed the QoL of transgender individuals receiving GAHT who did not undergo GAS and reported improved QoL, anxiety, and depression in transgender individuals receiving GAHT vs. those without hormone therapy. However, high-quality research on the impact of GAHT-related physical changes on QoL is still needed in the transgender population; likewise, a validated QoL instrument for the trans population is still lacking (28).

Therefore, the aims of this study were to assess perceived QoL in a sample of individuals with gender dysphoria before genital surgery, to compare QoL scores between trans women and men and to identify sociodemographic and physical characteristics related to GAHT that can contribute to their QoL.

## Materials and Methods

### Participants and Recruitment

This cross-sectional study evaluated transgender women and men recruited by availability sampling from the outpatient endocrine clinic of the Gender Identity Program at the Hospital de Clínicas de Porto Alegre (HCPA), Brazil. Patients over 18 years old with a confirmed diagnosis of gender dysphoria receiving medically prescribed GAHT for at least 6 months were consecutively included. Also included were trans men who had undergone mastectomy and trans women who had received breast augmentation surgery. Individuals who had undergone GAS (specifically genital surgery) or with uncontrolled clinical/psychiatric conditions at the time of the initial assessment were excluded.

Recruitment took place over a 12-month period between 2016 and 2017. A total of 135 transgender individuals were invited. Seventeen individuals with previous GAS (12.6%) and five who refused to participate (3.7%) were excluded. Therefore, 113 patients were enrolled and completed the study (60 trans women and 53 trans men).

### Sociodemographic Variables and Physical Examination

Sociodemographic and clinical data as well as hormone levels were collected from medical records: age, schooling, occupational status, marital status, smoking, alcohol use disorder (2), illicit drug use, sexually transmitted infections (STIs), physical activity levels, previous mastectomy (trans men), and presence of breast implants (trans women). Smoking status was categorized as current smoker, former smoker, or never smoker. Active individuals were those engaged in moderate-intensity physical activity for at least 150 min or in vigorous-intensity physical activity for 75 min per week, according to the World Health Organization (29) definition for people between 18 and 64 years of age. Individuals not meeting these criteria were considered sedentary.

Blood pressure was measured after a 10-min rest in a sitting position, with the feet on the floor and the arm supported at the level of the heart, using an automatic blood pressure monitor with an appropriate cuff for the arm diameter (Omron HEM 742, Rio de Janeiro, Brazil). Weight was measured in kilograms (kg) using an electronic anthropometric scale with a 100 g scale, with a capacity of 180 kg. All individuals were weighed barefoot and wearing an apron (Filizola Personal, São Paulo, Brazil). Height was measured in meters (m), using a stadiometer fixed to the wall (Tonelli E150A, Santa Catarina, Brazil). Body mass index (BMI) was calculated as weight in kilograms divided by squared height in meters (kg/m^2^) and was categorized as normal weight (18 to <25), overweight (25 to <30), and obesity (≥30). The degree of body hairiness was assessed using the Ferriman-Gallwey scale, which visually attributes a score from zero (no hair) to four to nine body areas, with scores summed to provide a total score. Trans men were stratified according to median Ferriman-Gallwey values into two groups: ≤ or >20 (30). The Tanner scale was used to assess the degree of breast development, with individuals categorized into two groups (Tanner < or ≥4) (31).

### Gender-Affirming Hormone Therapy

Trans women were prescribed oral estrogen (estradiol valerate, 2–4 mg/d) associated with antiandrogen (spironolactone 50–150 mg/d or cyproterone acetate 50–100 mg/d). For trans men, intramuscular testosterone cypionate was used (200 mg every 2–4 weeks). Dosages were compatible with the gender identity of individuals, and individualized according to clinical response and laboratory parameters (6). Upon arrival at our outpatient clinic and before a short washout period for clinical evaluation, 47 (78.3%) trans women and 19 (35.8%) trans men were already self-medicating with different hormone treatments and dosages.

### Quality of Life Assessment

QoL was assessed using a validated Brazilian Portuguese version of the WHOQOL-BREF questionnaire (32). WHOQOL-BREF is composed of 26 structured questions, of which two are general questions about QoL and 24 questions represent each facet of the WHOQOL-100, divided into four domains: physical, psychological, social relations, and environment (32, 33). The average score in each domain indicates the individual's perceived satisfaction with each QoL aspect. Each individual WHOQOL-BREF item was scored in a Likert scale from 1 (very dissatisfied /very bad) to 5 (very satisfied/very good), resulting in final scores on a scale of 4–20 for each domain. All scores are multiplied by 4 to be directly comparable with scores derived from the WHOQOL-100 (33). For interpretation purposes, all scores were transformed into a scale from 0 to 100 to allow comparisons between domains containing different numbers of items, as previously reported (15, 34). Higher scores (closer to 100%) indicate a better self-perceived QoL. The WHOQOL-BREF has been shown to be a reliable instrument for the assessment of QoL in the general population in several countries (33, 35). The questionnaire was administered by the interviewer during an outpatient visit scheduled after 6 months of GAHT. The answers referred to the last 2 weeks prior to the day of data collection.

### Statistical Analysis

Winpepi® was used for sample size calculation. Sample size was estimated based on a previous study, according to which the chance of higher QoL in the social domain was higher in trans men than trans women (15). Thus, considering a power of 80% and alpha of 5%, 100 trans individuals would be required to detect a difference of approximately 10% between WHOQOL-BREF domain scores between trans women and trans men (15). Variables with Gaussian distribution were assessed by the Shapiro-Wilk normality test. Continuous variables were expressed as mean and standard deviation and median and interquartile range for variables with non-Gaussian distribution. Categorical variables were expressed as frequency and percentage. Comparisons between two categories were analyzed using Student's *t*-test, and comparisons between more than two categories were analyzed by ANOVA. The chi-square (χ^2^) test was used for categorical variables. Data were analyzed using the Statistical Package for the Social Sciences, version 18.0 (SPSS Inc., Chicago, IL). *P* < 0.05 were considered statistically significant.

## Results

### Clinical and Sociodemographic Characteristics in Trans Women and Trans Men

Clinical, hormonal, and sociodemographic characteristics are shown in Table 1.

**Table 1:** Clinical, hormonal, and sociodemographic characteristics of transgender individuals.

**Variable**	**All (113)**	**Trans women (60)**	**Trans men (53)**	***p***
Age (years)	32.5 ± 9	34.1 ± 8.7	30.8 ± 9.2	0.053^a^
**Schooling—*****n*** **(%)**				
<9 years	28 (24.8)	14 (23.3)	14 (26.4)	0.424^b^
>9 years	85 (75.2)	46 (76.7)	39 (73.6)	
**Occupation—*****n*** **(%)**				
Employed	78 (69)	45 (75)	33 (62.3)	0.144^b^
Unemployed	27 (23.9)	15 (25)	12 (22.6)	
Student	8 (7.1)	–	8 (15.1)	
**Marital status—*****n*** **(%)**				
Stable relationship	48 (42.5)	25 (41.7)	23 (43.4)	0.853^b^
Single	65 (57.5)	35 (58.3)	30 (56.6)	
Gender-affirming hormone therapy (months)	21.81 ± 8.31	22.15 ± 8.57	21.43 ± 8.07	0.650^a^
**Previous hormone therapy—*****n*** **(%)**				
Yes	66 (58.4)	47 (78.3)	19 (35.8)	** <0.001**^**b**^
No	47 (41.6)	13 (21.7)	34 (64.2)	
**Physical activity—*****n*** **(%)**				
Active	27 (23.9)	12 (20)	15 (28.3)	0.302^b^
Sedentary	86 (76.1)	48 (80)	38 (71.7)	
BMI (kg/m^2^)	28 ± 5.4	27 ± 4.1	29 ± 6.4	0.077^a^
18 to <30—*n* (%)	75 (66.4)	44 (73.3)	31 (58.5)	** <0.001**^**a**^
≥30—*n* (%)	38 (33.6)	16 (26.7)	22 (41.5)	
Estradiol (pmol/L)	–	203 (135–331)	160 (127–254)	–
Testosterone (nmol/L)	–	0.92 (0.31–4.48)	16.63 (9.82–21.97)	
SHBG (nmol/L)	–	53 (33–90)	20 (16–30)	
SBP	118.3 ± 10.4	118.2 ± 10.8	118.5 ± 10.1	0.870^a^
DBP	78.7 ± 7.8	79.4 ± 8.5	77.9 ± 6.9	0.313^a^
**Smoking—*****n*** **(%)**				
Yes	13 (11.5)	8 (13.3)	5 (9.4)	0.726^b^
No	88 (77.9)	45 (75)	43 (81.2)	
Former smoker	12 (10.6)	7 (11.7)	5 (9.4)	
**Alcohol use disorder—*****n*** **(%)**				
Yes	2 (1.8)	2 (3.3)	–	–
No	106 (93.8)	56 (93.4)	50 (94.3)	
Former alcohol use disorder^1^	5 (4.4)	2 (3.3)	3 (5.7)	
**Illicit drug use—*****n*** **(%)**				
Yes	2 (1.8)	2 (3.3)	–	–
No	108 (95.6)	56 (93.4)	52 (98.1)	
Former drug use	3 (2.6)	2 (3.3)	1 (1.9)	
**HIV—*****n*** **(%) (110)**^**c**^				
Reactive	13 (11.8)	13 (22.4)	–	–
Non-reactive	97 (88.2)	45 (77.6)	52 (100)	
**HCV—*****n*** **(%) (108)**^**c**^				
Reactive	–	–	–	–
Non-reactive	108 (100)	56 (100)	52 (100)	
**HBsAg—*****n*** **(%) (103)**^**c**^				
Reactive	1 (1)	1 (1.8)	–	–
Non-reactive	102 (99)	54 (98.2)	48 (100)	
**VDRL—*****n*** **(%) (103)**^**c**^				
Reactive	10 (9.7)	10 (18.9)	–	–
Non-reactive	93 (90.3)	43 (81.1)	50 (100)	
**Breast surgical procedure—*****n*** **(%)**				
Yes	–	18 (30)	13 (24.5)	0.515^b^
No	–	42 (70)	40 (75.5)	
**Tanner (breast)—*****n*** **(%)**				
<4	–	29 (48.3)	–	–
≥ 4	–	31 (51.7)	–	
**Ferriman-Gallwey—*****n*** **(%)**				
≤20	–	–	23 (43.4)	–
>20	–	–	30 (56.6)	

*BMI, body mass index (18 to <30: normal weight and overweight, ≥30: obesity); SHBG, sex hormone binding globulin; SBP, systolic blood pressure; DBP, diastolic blood pressure; HIV, human immunodeficiency virus; HCV, hepatitis C virus; HBsAg, serology for hepatitis B; VDRL, venereal disease research laboratory for syphilis; Breast surgical procedure: breast implants for trans women and mastectomy for trans men*.

*Data expressed as mean and standard deviation, median and interquartile range or frequency and percentage*.

a *Student's t-test*.

b *Pearson's chi-square test*.

c *Number of individuals tested*.

1 *According to DSM-5 American Psychiatric Association. Bold values indicate statistical significance*.

The mean age of transgender individuals was 32.5 ± 9.0 years. Most participants (75.2%) had more than 9 years of schooling, 69% were employed, and 42.5% were in a stable relationship. This profile was similar in trans women and trans men. Mean blood pressure was within the reference values for normotensive adults, and 23.9% of the individuals were considered physically active, with no differences between the groups. Mean BMI was 28.0 ± 5.4 kg/m^2^, and obesity was observed in 33.6%. The rate of obesity was higher in trans men (41.5%) than in trans women (26.7%; *p* < 0.001; Table 1).

The prevalence of current smoking was 11.5%, and alcohol abuse and illicit drug use was reported by 1.8%. Thirteen participants were HIV-positive, and 10 were VDRL-reactive—all of them were trans women. Among HIV-positive patients, 92.0% were using antiretroviral drugs. The percentage of trans women who had a previous breast implant procedure was 30.0 and 24.5% of trans men had a mastectomy (Table 1).

Regarding GAHT formulations, 47 (78.3%) trans women were using estradiol valerate 2 mg/day, 8 (13.3%), 3 mg/day, and 5 (8.4%), 4 mg/day. With respect to antiandrogen drugs, 18 (30.0%) were using spironolactone 50 mg/day, 32 (53.3%), 100 mg/day and 4 (6.7%), 150 mg/day; 6 (10.0%) were using cyproterone acetate 50 mg/day. All trans men were using intramuscular testosterone cypionate 200 mg every 2–4 weeks. Median hormone levels were within the range for GAHT-treated persons, and a higher proportion of trans women (78.3%) reported previous irregular use of self-medicated hormone therapy than trans men (35.8%). The mean duration of medically prescribed GAHT was 21.81 ± 8.31 months.

### Comparisons of Perceived Quality of Life in Trans Women and Trans Men

The scores for the WHOQOL-BREF domains were presented on a scale of 0–100. In general, individuals had scores above 60%, without significant differences between trans women and trans men (Table 2).

**Table 2:** Mean WHOQOL-BREF scores in transgender individuals.

**Domain**	**All (113)**	**Trans women (60)**	**Trans men (53)**	***p***
Physical	68.23 ± 17.85	69.88 ± 17.13	66.37 ± 18.62	0.300
Psychological	63.40 ± 17.15	64 ± 15.26	62.73 ± 19.2	0.698
Social relations	69.61 ± 20.94	70.13 ± 18.55	69.02 ± 23.53	0.779
Environment	60.23 ± 14.55	59.73 ± 14.61	60.79 ± 14.60	0.704
Quality of life	73.23 ± 17.82	75.83 ± 16.57	70.28 ± 18.86	0.099

*Data expressed as mean and standard deviation; Domains with scores from 0 to 100*.

*Student's t-test*.

In both groups, the lowest QoL score was recorded in the environmental domain (trans women = 59.73 ± 14.61, trans men = 60.79 ± 14.60), followed by the psychological and physical domains (64 ± 15.26 and 69.88 ± 17.13 for trans women; 62.73 ± 19.20 and 66.37 ± 18.62 for trans men, respectively). The highest score was recorded in the social relations domain: 70.13 ± 18.55 for trans women and 69.02 ± 23.53 for trans men. Mean overall QoL was 75.83 ± 16.57 for trans women and 70.28 ± 18.86 for trans men.

### Quality of Life Scores According to Sociodemographic and Clinical Characteristics

#### Transgender Women

When comparing sociodemographic data categories and clinical characteristics according to WHOQOL-BREF domains, the social relations domain score was significantly higher in trans women in a stable relationship than in single ones (76.66 ± 10.75 vs. 65.47 ± 21.49; *p* = 0.011), and in those with BMI ≥30 (78.64 ± 13.25 vs. 67.04 ± 19.35; *p* = 0.031). In addition, trans women with Tanner stage 4 or greater breast development had a significantly higher mean physical domain score (75.49 ± 13.95 vs. 64.63 ± 18.35, *p* = 0.013). No significant differences in QoL scores were observed between trans women with and without breast implants. Among those without breast implants, 31% had a Tanner score ≥4. QoL scores were similar in different occupation, education, and HIV categories (Table 3).

**Table 3:** Mean WHOQOL-BREF scores in trans women according to sociodemographic and clinical characteristics (*N* = 60).

	**WHOQOL-BREF domains**									
	**Physical**	***p***	**Psychological**	***p***	**Social relations**	***p***	**Environment**	***p***	**Quality of life in general**	***p***
**Sociodemographic characteristics**										
**Marital status**										
Single	68.97 ± 17.03	0.634	62.8 ± 14.81	0.307	65.47 ± 21.49	**0.011**	60.17 ± 14.44	0.786	74.28 ± 14.2	0.422
Stable relationship	71.14 ± 17.55		66.4 ± 15.84		76.66 ± 10.75		59.12 ± 15.11		78.00 ± 19.52	
**Occupation**										
Employed	72.38 ± 14.87	0.111	65.33 ± 13.95	0.244	69.62 ± 18.47	0.716	61.66 ± 14.12	0.070	75.55 ± 14.09	0.862
Unemployed	62.38 ± 21.50		60.00 ± 18.61		71.66 ± 19.36		53.95 ± 15.00		76.66 ± 23.08	
**Education level**										
<9 years	65.05 ± 20.73	0.232	57.50 ± 17.4	0.068	72.02 ± 12.05	0.668	58.70 ± 18.35	0.765	77.67 ± 22.56	0.712
>9 years	71.35 ± 15.85		65.97 ± 14.16		69.56 ± 20.20		60.05 ± 13.50		75.27 ± 14.55	
**Physical activity**										
Active	73.80 ± 17.81	0.379	63.75 ± 14.79	0.950	70.83 ± 20.87	0.886	57.55 ± 9.83	0.567	76.04 ± 16.39	0.962
Sedentary	68.89 ± 16.99		64.06 ± 15.52		69.96 ± 18.17		60.28 ± 15.61		75.78 ± 16.79	
**HIV**										
Reactive	70.32 ± 18.34	0.953	68.86 ± 12.77	0.221	75.00 ± 17.34	0.260	61.05 ± 15.28	0.760	75.96 ± 17.27	0.929
Non-reactive	70.63 ± 15.69		63.11 ± 15.19		68.33 ± 18.93		59.65 ± 14.28		76.38 ± 14.65	
**Clinical characteristics**										
**Tanner stage**										
<4	64.63 ± 18.35	**0.013**	60.80 ± 16.98	0.94	68.54 ± 19.21	0.497	58.36 ± 14.99	0.457	72.54 ± 17.5	0.117
≥4	75.49 ± 13.95		67.41 ± 12.57		71.83 ± 18.01		61.2 ± 14.3		79.31 ± 15.04	
**Breast implants**										
Yes	67.6 ± 17.71	0.116	62.97 ± 15.73	0.118	70.83 ± 19.32	0.662	58.63 ± 15.33	0.374	75.59 ± 16.78	0.374
No^a^	75.19 ± 14.82		66.38 ± 14.22		68.51 ± 17.04		62.32 ± 12.79		76.38 ± 16.54	
**BMI**										
18 to <30	69.23 ± 18.99	0.633	62.15 ± 16.26	0.068	67.04 ± 19.35	**0.031**	58.80 ± 15.54	0.417	75.85 ± 17.34	0.988
≥30	71.65 ± 10.79		69.06 ± 10.98		78.64 ± 13.25		62.30 ± 11.71		75.78 ± 14.76	

*Data expressed as mean and standard deviation; domains with scores from 0 to 100*.

a *Tanner ≥4 in 31%; Student's t-test. Bold values indicate statistical significance*.

#### Transgender Men

Significantly higher scores were observed in trans men in a stable relationship than in single ones in the following domains: physical (75.73 ± 14.86 vs. 60.71 ± 19.44; *p* = 0.010), psychological (69.13 ± 14.35 vs. 57.83 ± 21.15; *p* = 0.032), and social relations (75.72 ± 17.57 vs. 63.88 ± 26.38; *p* = 0.049). Also, increased body hair (Ferriman-Gallwey score >20) was associated with higher QoL scores in the physical (71.78 ± 16.81 vs. 59.31 ± 18.85; *p* = 0.014), psychological (67.83 ± 15.62 vs. 56.08 ± 21.63; *p* = 0.026), and social relations domains (74.44 ± 18.81 vs. 61.95 ± 27.38; *p* = 0.049). Being physically active was associated with higher physical (74.04 ± 11.41 vs. 63.34 ± 20.12; *p* = 0.019), psychological (70.66 ± 17.40 vs. 59.60 ± 19.18; *p* = 0.049), social relations (78.33 ± 19.10 vs. 65.35 ± 24.31; *p* = 0.048), and environment domain scores (70.00 ± 10.21 vs. 57.15 ± 14.56; *p* = 0.003) when compared to being sedentary. Being in a stable relationship was also associated with a higher general WHOQOL-BREF score (76.08 ± 15.46 vs. 65.83 ± 20.21; *p* = 0.049; Table 4).

**Table 4:** Mean WHOQOL-BREF scores in trans men according to sociodemographic and clinical characteristics (*N* = 53).

	**WHOQOL-BREF domains**									
	**Physical**	***p***	**Psychological**	***p***	**Social relations**	***p***	**Environment**	***p***	**Quality of life in general**	***p***
**Sociodemographic characteristics**										
**Marital status**										
Single	60.71 ± 19.44	**0.010**	57.83 ± 21.15	**0.032**	63.88 ± 26.38	**0.049**	58.85 ± 15.26	0.274	65.83 ± 20.21	**0.049**
Stable relationship	75.73 ± 14.86		69.13 ± 14.35		75.72 ± 17.57		63.31 ± 13.59		76.08 ± 15.46	
**Occupation**^**a**^										
Employed	72.07 ± 16.49^a^	**0.004**	67.57 ± 16.06^a^	**0.010**	70.20 ± 19.65	0.348	64.01 ± 13.73	0.071	72.34 ± 19.70	0.378
Unemployed	52.67 ± 19.56^b^		49.16 ± 21.08^b^		61.11 ± 31.04		52.86 ± 13.87		63.54 ± 18.81	
Student	63.39 ± 15.71^ab^		63.12 ± 20.86^ab^		76.04 ± 25.75		59.37 ± 16.10		71.87 ± 14.56	
**Education level**										
<9 years	63.77 ± 23.09	0.548	58.92 ± 21.49	0.392	72.61 ± 25.19	0.511	56.91 ± 15.02	0.251	75.00 ± 16.26	0.280
>9 years	67.30 ± 17.00		64.10 ± 18.42		67.73 ± 23.11		62.17 ± 14.38		68.58 ± 19.63	
**Physical activity**										
Active	74.04 ± 11.41	**0.019**	70.66 ± 17.40	**0.049**	78.33 ± 19.10	**0.048**	70.00 ± 10.21	**0.003**	75.00 ± 13.36	0.257
Sedentary	63.34 ± 20.12		59.60 ± 19.18		65.35 ± 24.31		57.15 ± 14.56		68.42 ± 20.49	
**Clinical characteristics**										
**Ferriman-Gallwey**										
≤20	59.31 ± 18.85	**0.014**	56.08 ± 21.63	**0.026**	61.95 ± 27.38	**0.049**	58.55 ± 13.82	0.335	69.02 ± 19.16	0.674
>20	71.78 ± 16.81		67.83 ± 15.62		74.44 ± 18.81		62.50 ± 15.17		71.25 ± 18.90	
**Mastectomy**										
Yes	69.78 ± 20.34	0.453	63.07 ± 24.28	0.942	73.07 ± 25.03	0.480	67.06 ± 11.45	0.074	75.96 ± 16.50	0.215
No	65.26 ± 18.71		62.62 ± 17.61		67.70 ± 23.20		58.75 ± 15.04		68.43 ± 19.04	
**BMI**										
18 to <30	70.04 ± 17.70	0.089	64.83 ± 19.16	0.349	70.96 ± 21.06	0.481	61.89 ± 14.64	0.518	71.37 ± 18.31	0.623
≥30	61.20 ± 19.06		59.77 ± 19.30		66.28 ± 26.90		59.23 ± 14.73		68.75 ± 19.95	

*Data expressed as mean and standard deviation; domains with scores from 0 to 100*.

*Student's t-test*.

a *Two-way ANOVA*.

b,ab *Different superscript letters in each row indicate which groups differ statistically for the specific variable. Bold values indicate statistical significance*.

Employment was related to higher QoL scores. Students had intermediate scores vs. the other occupation categories in the physical (*p* = 0.004) and psychological (*p* = 0.010) domains.

No significant differences in QoL scores were observed regarding prior mastectomy, BMI, and education level (Table 4).

## Discussion

In the present study, QoL scores were similar in trans women and trans men receiving GAHT. Among trans women, higher QoL scores were associated with breast development, being in a stable relationship, and greater BMI. In trans men, QoL was associated with increased body hair, being in a stable relationship, being physically active, and being employed. While a few previous studies have shown a general benefit of GAHT to QoL in transgender individuals (15, 18, 24, 28, 36), to the best of our knowledge this is the first study to specifically evaluate QoL in association with GAHT-related clinical characteristics in trans men and trans women not submitted to GAS (except for mastectomy/breast augmentation surgery in some trans men and women, respectively).

The mean BMI observed in trans women was 27 kg/m^2^. Obese trans women had a better score in the social relations domain when compared to trans women with normal BMI and overweight. Recent studies have demonstrated that GAHT is associated with significant body fat gain and reduced muscle mass (36, 37). Indeed, a multicenter study including 179 trans women found an increase of 42% body fat in the legs, 18% in the android region and 34% in the gynoid region (23). Thus, it is possible that the higher social relations score observed in our obese patients is related to a sense of well-being produced by a more feminine body fat distribution.

In relation to trans men, the majority in our study had BMI values compatible with overweight or obesity. However, there was no significant difference in the QoL scores between obese and non-obese categories. Recent studies have shown that GAHT for trans men is associated with increased BMI. In a systematic review of 13 studies, we have previously detected an increase of 1.3–11.4% in BMI with the use of testosterone (38). It is likely that this increase in BMI occurs at the expense of increased muscle mass associated with the anabolic effect of testosterone (37).

In the present study, we observed that being in a stable relationship was significantly associated with the social relation domain in trans women and a better score in the physical, psychological, social relations, and general QoL domains in trans men, which is in agreement with other studies in the literature (18, 21, 39). Motmans et al. (21) assessed 139 individuals and observed that 52.5% were in a stable relationship and showed better QoL scores in physical functioning, general health perceptions, and social functioning in comparison with single individuals. Conversely, in a study with 209 Chinese trans women, not having regular partner was positively associated with the mental and physical component of QoL (40). The higher prevalence of depression described by previous studies in the group of Chinese trans women in a stable relationship might be explained by the fear of losing their partners or suffering discrimination from them (41).

We identified that most individuals in our sample had more than 9 years of formal education. This subgroup had better QoL scores, with no significant difference between trans women and trans men. In terms of occupation, while most of our trans women were employed, no difference was found in QoL between employed and unemployed individuals. In trans men, we observed that employed individuals had better scores in the physical and psychological domains when compared to unemployed individuals or students. In the literature, data on education are conflicting (18, 20), but previous studies suggest that being employed is associated with better QoL in trans individuals (15, 18). In the study of Valashany and Janghorbani (20), with 71 individuals who were employed or worked on their own, most with secondary or higher education levels, a significant relationship was observed between education and subscales of emotional well-being and social function, between economic status and physical function subscale, and between employment status and physical and social function. In another study using the Short Form Health Survey (SF-36) with 61 trans individuals, most of whom were employed and had more than 12 years of schooling, no significant difference was observed in terms of education; however, those with employment had higher QoL scores in the physical function and vitality domains (18). Similarly, it was observed in the study of Gómez-Gil (15) in which 61% of the individuals were employed or studying, having an occupation had a positive association with the physical domain, social relation, environment, and general QoL scores, but no significant difference was observed in relation to the level of education.

Regarding physical characteristics, increased breast development was significantly associated with a higher score in the physical domain in trans women. In a study assessing body uneasiness and psychiatric symptoms in transgender individuals, a decrease in body discomfort was observed in trans women who used GAHT compared to those who did not use hormone therapy. The body characteristics that caused greater dissatisfaction were presence of body hair, smell, arms, thorax, buttocks, and eyes (42). Interestingly, no significant difference was found in quality of life between trans women with and without breast implants in the present study. Similarly, in a study conducted with 39 trans women, there was no difference in quality of life scores in trans women regarding breast implants (21). Breast development is generally perceived from 6 months to 2 years after the onset of hormone therapy, being different in each subject (6). In some cases, surgical intervention is necessary, but in other cases the individuals are satisfied with the size of their breast after GAHT.

In the present study, we found no significant differences between trans men with mastectomy and QoL, which differs from the results of other studies (8, 43). Newfield et al. (8) evaluated 136 trans men submitted to mastectomy and observed higher QoL scores in these individuals when compared to patients who had not performed mastectomy. Mastectomy appears to improve the identity of trans men, increasing self-esteem and the confidence to take off their T-shirts in favorable environments (8).

Trans men with increased body hair had higher scores in the physical, psychological, and social domains. In fact, beard growth can cause a significant change in the way trans men are perceived. Phenotypic alterations seem to be positively associated with amelioration of body image by individuals in GAHT, and this may contribute to significant improvement in interpersonal relations (36).

Literature data are limited regarding the *status* of physical activity in transgender individuals. In trans men, being physically active was significantly associated with a higher score in the physical, psychological, social relations, and environment domains. In a British study with a small sample size, transgender men were found to be less active despite being motivated to exercise to increase body satisfaction and congruence with the desired gender (44). These individuals reported that they lacked safe spaces for physical activity and adequate facilities to change clothing. They also mentioned body dissatisfaction and the concern that they would not be accepted by others (44). The same authors also reported that transgender individuals were less engaged in physical activity than cisgender individuals. However, using GAHT was linked to increased engagement in physical activity and greater body satisfaction when compared to not using GAHT (45).

In our study, there was no significant difference in the QoL scores in relation to HIV serology, which is in accordance with the literature (34). Most patients, however, were being treated with antiretroviral drugs. Trans women present higher prevalence of STIs, possibly due to social, biological, and behavioral factors (46). A meta-analysis with 11,066 trans women from 15 countries showed a prevalence of HIV infection of 19.1%, with an odds ratio of 48.8 (95% CI 21.2–76.3) for trans women being infected compared with adults in reproductive age (47).

The overall QoL score was similar between trans men and trans women. This was also observed by another study with 61 trans individuals, in which no differences were observed in QoL scores between trans men and trans women (18). Conversely, a study with 94 trans individuals reported better QoL scores in trans men in relation to trans women, especially in physical functioning and general health perception (21). Finally, a meta-analysis with 14 studies and 1,950 individuals revealed that transgender individuals showed worse QoL related to mental health when compared to the general population. However, those results did not remain significant in a second analysis focusing only on GAHT participants (48).

The present study has strengths, including the presentation of novel data on Brazilian transgender individuals, a less well-represented population in studies about factors associated with QoL. Also, the sample only included individuals who met DSM-5 criteria for gender dysphoria and were in regular use of GAHT. Limitations of this study include the lack of a control group and the relatively small sample size, precluding complementary analyses. In addition, further studies on body composition are needed in order to deepen the understanding of findings related to BMI.

## Conclusion

Data from this study suggest that GAHT-related physical characteristics (breast development in trans women and increased body hair in trans men) are similarly associated with higher QoL scores. Stable relationships are also associated positively with higher QoL in both groups. Regarding trans men, being physically active and being employed contributed to better QoL. Further studies with transgender people from other regions with distinct socio-cultural traits could confirm and expand the present results. Healthcare providers may take these factors into consideration when planning interventions to improve QoL in transgender individuals.

## Data Availability Statement

The raw data supporting the conclusions of this article will be made available by the authors, without undue reservation.

## Ethics Statement

The studies involving human participants were reviewed and approved by CAAE 79656117.5.0000.5327. The patients/participants provided their written informed consent to participate in this study.

## Author Contributions

ES, TF, and PS were involved in the conception and design of the study, contributed to analysis and interpretation of data, and drafted the manuscript. ES, TF, and RA contributed to data collection. ML revised the manuscript for intellectual content. All the authors read and approved the final manuscript.

## Conflict of Interest

The authors declare that the research was conducted in the absence of any commercial or financial relationships that could be construed as a potential conflict of interest. The handling editor is currently co-organizing a Research Topic with one of the author ML, and confirms the absence of any other collaboration.

## Publisher's Note

All claims expressed in this article are solely those of the authors and do not necessarily represent those of their affiliated organizations, or those of the publisher, the editors and the reviewers. Any product that may be evaluated in this article, or claim that may be made by its manufacturer, is not guaranteed or endorsed by the publisher.
